# Effect of electromagnetic field on abortion: A systematic review and meta-analysis

**DOI:** 10.1515/med-2021-0384

**Published:** 2021-11-03

**Authors:** Masumeh Ghazanfarpour, Zahra Atarodi Kashani, Reza Pakzad, Fatemeh Abdi, Fatemeh Alsadat Rahnemaei, Pouran Akhavan Akbari, Nasibeh Roozbeh

**Affiliations:** Student Research Committee, Kerman University of Medical Sciences, Kerman, Iran; Iranshahr University of Medical Sciences, Iranshahr, Iran; Faculty of Health, Ilam University of Medical Sciences, Ilam, Iran; Mother and Child Welfare Research Center, Hormozgan University of Medical Sciences, Bander Abbas, Iran; School of Nursing and Midwifery, Alborz University of Medical Sciences, Karaj, Iran; Student Research Committee, Nursing and Midwifery Faculty, Shahid Beheshti University of Medical Sciences, Tehran, Iran; Department of Midwifery, Nursing and Midwifery Faculty, Ardabil University of Medical Sciences, Ardabil, Iran

**Keywords:** electromagnetic field, abortion, miscarriage, radiofrequency waves

## Abstract

**Background:**

The increasing use of new technologies by pregnant women inevitably exposes them to the risks of the electromagnetic fields (EMFs). According to the World Health Organization, EMFs are the major sources of pollutants which harm human health. This study was aimed to evaluate the effects of EMF exposure on abortion.

**Methods:**

Web of Science, Cochrane Library, MEDLINE, PubMed, EMBASE, Scopus, and Google Scholar were searched until 2021. Pooled odds ratio (OR) with 95% confidence interval (CI) was estimated using a random-effects model. Heterogeneity was explored using Cochran’s Q test and *I* ^2^ index. A meta-regression method was employed to investigate the factors affecting heterogeneity between the studies. The Newcastle-Ottawa scale was used to assess the credibility of the studies.

**Results:**

Eligible studies (*N* = 17) were analyzed with a total of 57,693 participants. The mean maternal age (95% CI) was 31.06 years (27.32–34.80). Based on meta-analysis results, the pooled estimate for OR of EMF with its effects was 1.27 (95% CI: 1.10–1.46). According to the results of meta-regression, sample size had a significant effect on heterogeneity between studies (*p*: 0.030), but mother’s age and publication year had no significant effect on heterogeneity (*p*-value of bothwere >0.05). No publication bias was observed.

**Conclusion:**

Exposure to EMFs above 50 Hz or 16 mG is associated with 1.27× increased risk of abortion. It may be prudent to advise women against this potentially important environmental hazard. Indeed, pregnant women should receive tailored counselling.

## 1 Introduction

Radiofrequency (RF) waves are a type of non-ionizing radiation with short-wavelength radio and electromagnetic waves, which are at the low-energy end of the electromagnetic spectrum. RF waves have lower energy than some other types of non-ionizing radiation, but they have higher energy than extremely low-frequency (ELF) radiation [1]. Ionizing waves include ultraviolet [2] and low frequency electromagnetic waves such as X-rays and gamma rays [3].

When RF radiation is absorbed by the body in large enough amounts, it would produce heat [4]. Humans are exposed to electromagnetic fields (EMFs) produced not only by natural phenomena such as the earth’s magnetic field (MF) or lightning, but also by human activities, such as the use of power lines and electrical appliances [5]. The use of new technology (such as cell phone, Internet, radio, and television] requires integrated telecommunication antenna and thus, the effects and outcomes of the waves produced by them are a hazard to human health. The effects of these radiations on the vulnerable population such as children, the elderly, and pregnant women are different. Cell phone is a major source of electromagnetic waves, and the use of it has grown exponentially within the past few years [6].

EMFs are generally believed to have no relevant non-thermal effects on cells, tissues, and living organisms [7]. According to the World Health Organization (WHO), EMFs are the major sources of pollutants which harm human health [8]. Both pregnant women and fetuses are vulnerable because EMFs are in interaction with cells of embryo’s development. Short-wavelength electromagnetic waves can damage the placental barrier. The membrane prevents the transfer of substances between blood, and this fact indicates that pregnant women should not use cell phone except in case of emergency [9].

Spontaneous abortion is the most common complication of early pregnancy. Pregnancy loss before the 20th week of gestation can be defined as spontaneous abortion, or miscarriage. More than 80% of the miscarriages happen in the first 12 weeks of pregnancy. Approximately two-thirds of early miscarriages are clinically asymptomatic. Fetal, maternal, and paternal factors play an important role in the abortion. Chromosomal abnormality is considered as the most common cause of spontaneous abortion. Environmental, developmental, and medical factors should be considered for maternal abortion. X-ray is one of the factors affecting abortion [10,11].

Some of the reported risk factors for early miscarriage include using a cell phone or computer for more than 6 h a day [12] in addition to living in urban areas in comparison with living in rural areas and the residence within 100 m from mobile phone base stations. The gradual increase in the number of the abortions clinically recognized and the increase in electricity consumption over the past century are not taken into consideration. Increased exposure to each location occurred gradually over time and there was simultaneous improvement in prenatal care [13]. Electric fields in the face of different conductivity cells can significantly generate the heating field and get attenuated. Unlike the external electric fields, the MFs are unperturbed by human body and penetrate without attenuation. The human body is transparent to the MF because there is no magnetic material in the human body. Variable MFs generate electric fields in the human body through a mechanism known as the Faraday Effect [14].

One in four US women will have had an abortion [15]. Long Interspersed LINE-1 Element-1 retrotransposonsor endogenous mutations that can alter gene expression are a major cause of an early abortion in humans [16]. Some of the studies put emphasis on the impact of medium frequency EMF on early pregnancy loss [17]. Spontaneous abortion is reported to be linked with the increased use of electrically heated beds and blankets (which are sources of ELF-MFs) [18]. The non-thermal effects of RF-EMF on the treatment of the human colon cancer cell lines showed that these waves induce ion fluxes which could potentially cause relevant disequilibrium in most ions that may adversely influence the proliferation of cancer cells. Available preclinical and clinical data support the existence of non-thermal membrane/cellular injuries caused by RF-EMF; however, we would need further research [19].

Given that the increasing use of new technologies by pregnant women inevitably exposes them to the risks of the EMF, therefore it is necessary to recognize the unknown adverse effects of these waves for the pregnant women and their fetuses. Pregnant women are exposed to the EMFs for hours during the day during pregnancy. By recognizing the adverse effects of EMF exposure on the pregnant women and their fetuses and informing them, the adverse effects of EMF exposure can be prevented or their potential risks can be reduced. A few studies have investigated the effects of EMF exposure on abortion in pregnant women, and there is no general agreement on the effects of EMF exposure on abortion. On the other hand, the epidemiologic results about the possible adverse effects of low frequency EMF on the fertility are controversial. Therefore, the aim of this systematic review and meta-analysis was to investigate the effects of EMF exposure on abortion.

## 2 Materials and methods

Preferred Reporting Items for Systematic Reviews and Meta-Analyses (PRISMA) guidelines were observed in the report of the study [20]. The protocol of the study was registered in the International Prospective Register of Systematic Reviews (PROSPERO) at the National Institute for Health Research (No: CRD42021234492).

### 2.1 Search strategy

An updated literature search for studies published until 2021 was conducted in Web of Science, Cochrane Library (Wiley), MEDLINE (Ovid), Pub Med, EMBASE (Ovid), CINAHL (EBSCO), Scopus, Google Scholar, and Clinicaltrial.gov with no restrictions on the language of publication (Table 1).

**Table 1:** Search Strategy for systematic review

ID	Search term
1.	“electromagnetic fields”[MeSH Terms] OR “electromagnetic field”[Title/Abstract] OR “magnetic field”[Title/Abstract] OR “electromagnetic wave”[Title/Abstract] OR “EMF”[Title/Abstract] OR “EMW”[Title/Abstract] OR “cell phone”[Title/Abstract] OR “cellphone”[Title/Abstract] OR “car phone”[Title/Abstract] OR “cellular phone”[Title/Abstract] OR “cellular telephone”[Title/Abstract] OR “mobile phone”[Title/Abstract] OR “mobile telephone”[Title/Abstract] OR “Telephone”[Title/Abstract] OR “Mobile”[Title/Abstract] OR “heated water”[Title/Abstract] OR “electric blanket”[Title/Abstract] OR “wire codes”[Title/Abstract] OR “X-rays”[Title/Abstract] OR “computer terminal”[Title/Abstract] OR “video display terminal”[Title/Abstract] OR “VDTs”[Title/Abstract] OR “Microwave”[Title/Abstract] OR “microwaves”[MeSH Terms] OR “radiofrequency”[Title/Abstract] OR “RF”[Title/Abstract] OR “RF-EMFs”[Title/Abstract]
2.	“abortion, spontaneous”[MeSH Terms] OR “spontaneous abortion”[Title/Abstract] OR “Abortion”[Title/Abstract] OR “Miscarriage”[Title/Abstract] OR “fetal loss”[Title/Abstract] OR “early pregnancy loss”[Title/Abstract] OR “early pregnancy losses”[Title/Abstract]
3.	#1 AND #2

### 2.2 Inclusion and exclusion criteria

The study’s inclusion criteria were as follows: women with intrauterine pregnancies, singleton pregnancy, and spontaneous conception without the use of the assisted reproductive techniques. Exclusion criteria for the study were as follows: studies with no access to their full texts, correspondences and letters to editor, a history of chronic diseases (diabetes, hypertension, cardiovascular, and liver diseases) [21], genetic disorders, vaginal bleeding, and birth defects in previous pregnancies, and cigarette smoking. Respectively Participants, Exposure, Comparators, Outcomes, Study Design criteria were pregnant women (P), electromagnetic field (E), Control group (C), Abortion (O), Cohort, Case control (S).

### 2.3 Study selection

**Figure 1:**
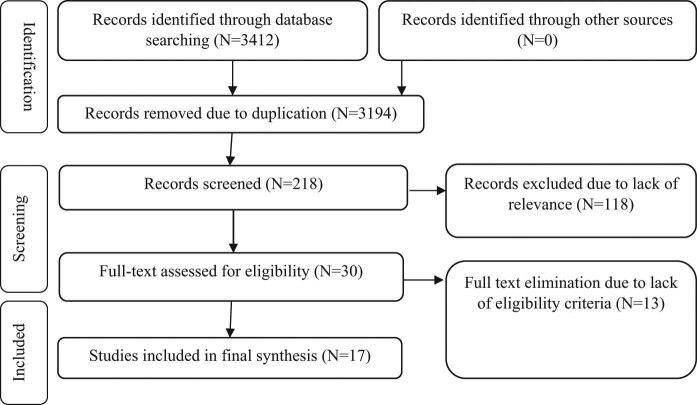
PRISMA Flowchart of selected studies.

The initial search yielded 3,412 results. The eligibility of these studies was independently evaluated by two authors and any disagreements were resolved by consensus. In the first stage, 3,194 studies were excluded due to being irrelevant or duplicated. After reviewing the titles and abstracts of the remaining studies, 188 articles were excluded from the study. In the evaluation of the full texts, 13 out of the remaining 30 articles were excluded due to being ineligible. Finally, a total of 17 eligible articles were reviewed (Figure 1).

### 2.4 Quality assessment

Quality of each study was assessed according to the Newcastle-Ottawa scale (NOS). A maximum of ten stars can be given to each study based on the NOS. A maximum of five stars can be given to the selection (such as sample size, non-respondents, and ascertainment of the exposure). A maximum of two stars can be given to the comparability (such as the study control for the most important factor). A maximum of three stars can be given to the outcome (such as outcome of the assessment and statistical test). Studies with a score of nine or ten stars are considered to be of high quality, and studies with a score of seven or eight stars are considered to be of medium quality, and studies scoring less than six stars are considered to be of low quality [22].

### 2.5 Data extraction

Two authors independently performed the study selection and validity assessment, and resolved any disagreements by consulting a third researcher. Criteria was examined in the study included the first author name, publication year, study design, sample size, region, age, gestational age, electromagnetic field, results, and quality score.

## 3 Statistical analysis

All the statistical analysis was performed using Stata software version 14.0 (College Station, Texas). For each study, odds ratio (OR) and 95% confidence interval (CI) of miscarriage were extracted. For some studies that reported hazard ratio or risk ratio, based on below formulas, these values were converted to OR:

\begin{array}{c}\text{RR}=(1-{e}^{\text{HR}\ast \mathrm{ln}(1-r)})/r\\ \text{OR}=((1-p)\ast \text{RR})/(1-\text{RR}\ast p),\end{array}]

where RR is the relative risk, HR is the hazard ratio; *r* is the rate for the reference group, and *p* is the incidence of the outcome of interest in the non-exposed group (based on baseline data).

Then, pooled OR was calculated by “Metan” command. Heterogeneity was determined using Cochran’s Q test of heterogeneity, and the *I* ^2^ index was used to quantify heterogeneity. In accordance with the Higgins classification approach, *I* ^2^ values above 0.7 were considered as high heterogeneity. To estimate the pooled OR for miscarriage, the fixed-effect model was used, and when the heterogeneity was greater than 0.7, the random effects model was used. The meta-regression analysis was used to examine the effect of age, sample size, and publication date as factors affecting heterogeneity among studies [23]. The “Metabias” command was used to check for publication bias, and if there was any publication bias, the pooled OR was adjusted with the “Metatrim” command using the trim-and-fill method [24,25]. *p*-values of 0.05 were regarded as statistically significant.

## 4 Results

Finally, 17 studies with a total sample size of 57,693 participants included in the study were selected (Table 1). The flowchart of this selection process is shown in Figure 1. Studies examined were published from 1988 to 2018, most of them were done in the USA with 6 (26.1%) studiesand mean maternal age (95% CI) was 31.06 years (27.32–34.80).

## 5 Pooled estimate of OR

**Figure 2:**
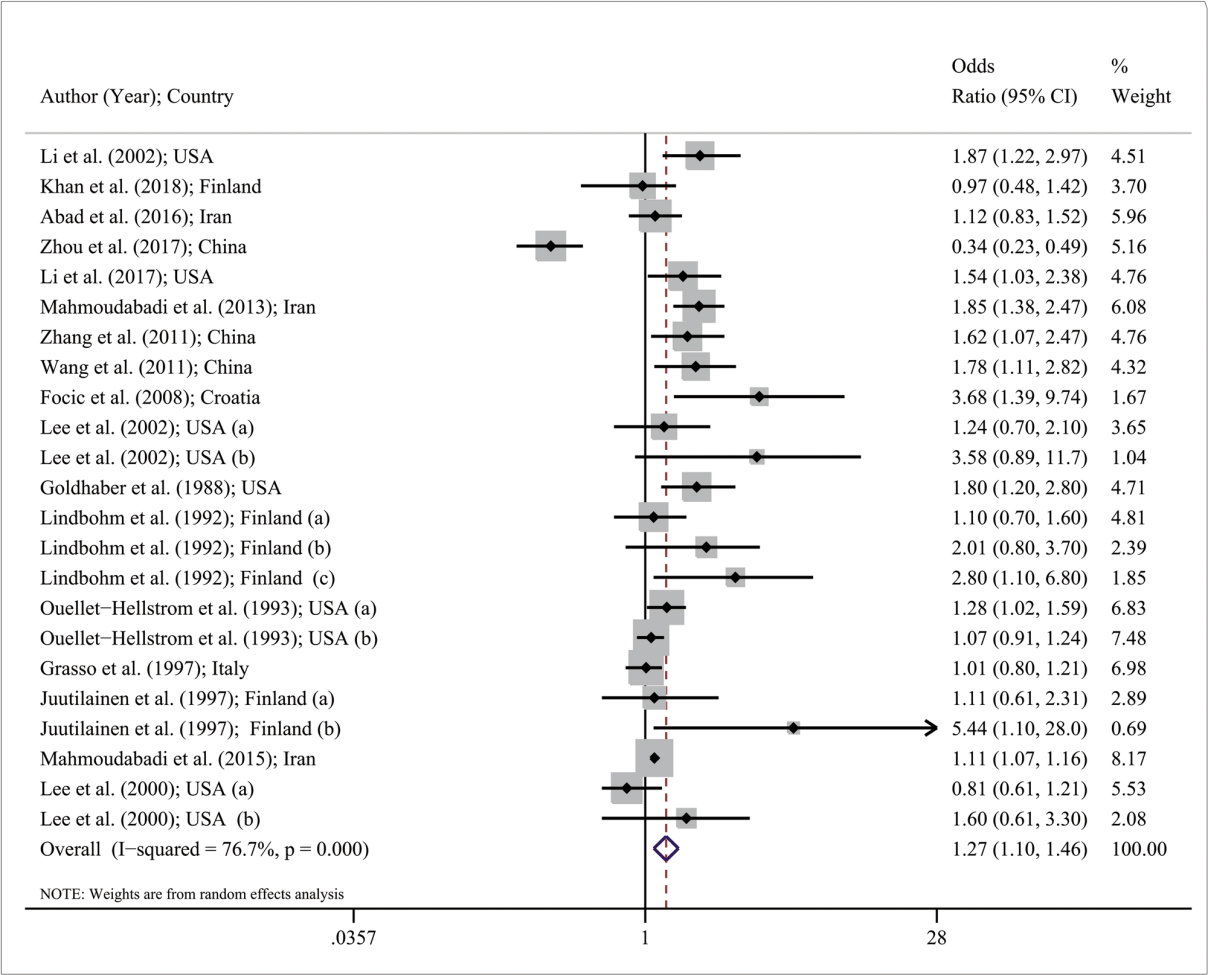
Forest plot for odds ratio of miscarriage based on random effects model. The midpoint of each line segment shows the odds ratio of miscarriage, the length of the line segment indicates the 95% confidence interval in each study, and the diamond mark illustrates the pooled odds ratio.

Figure 2 shows the forest plot for OR of abortion in the included studies. The minimum and maximum OR of abortion in China were reported by Zhou et al. was (OR: 0.34; 95% CI: 0.23–0.49) and by Juutilainen et al. was (OR: 5.40; 95% CI: 1.10–28.01), respectively. As shown in Figure 2, the pooled estimate for OR was 1.27 (95% CI: 1.10–1.46) using random effects model approach. This means that in overall, the exposure to EMF was a disfavor for miscarriage so that the odds ratio of miscarriage due to EMF exposure was 1.27× greater than that of control/non-exposed groups.

## 6 Heterogeneity and meta-regression results

**Figure 3:**
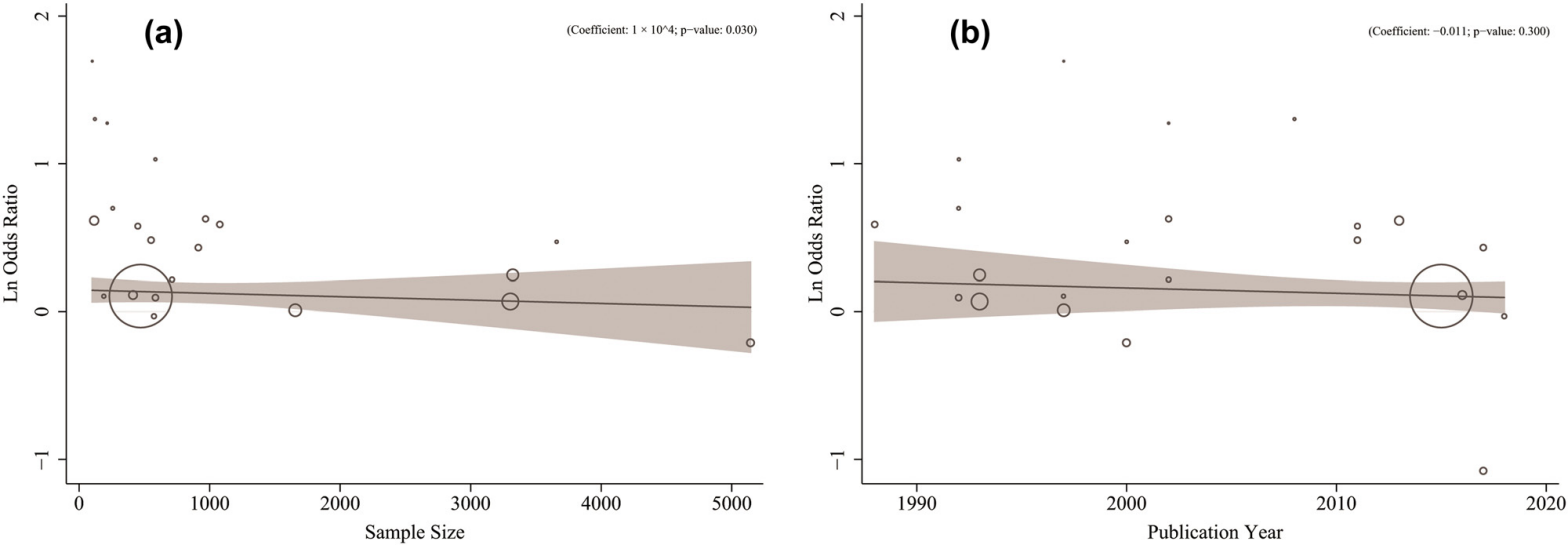
Association between odds of miscarriage caused by EMF with the sample size (a) and publication year (b) using meta-regression. Size of the circles indicates sample magnitude. There was significant relationship between Ln OR of miscarriage with sample size; but did not change markedly during the study years in this survey.

According to Cochran’s *Q* test of heterogeneity, there was a significant heterogeneity among studies (*p* < 0.001). The heterogeneity amount was 76.7% based on the *I* ^2^ index, indicating high heterogeneity. Table 2 presents the results of the univariate meta-regression. There were significant associations between results from studies and sample size (Coefficient: 1 × 10^4^; *p*-value: 0.030). Mother’s age (Coefficient: 0.051; *p*-value: 0.537) and publication year (Coefficient: −0.011; *p*-value: 0.300) had no significant effect on heterogeneity (Table 3 and Figure 3).

**Table 2:** Overview of all studies included in systematic review

Author (year)	Study design	Sample size	Region	Age (year)	GA (week)	EMF	Results	QS
Khan et al. (2018) [26]	Cohort	4,157 (574)	Finland	18.4–39 (27.2)	26.7–42.3	EAS devices	No excess risk of miscarriage were observed between cashiers working in grocery stores with and without EAS systems (OR: 0.97; 95% CI: 0.48–1.93)	9
Zhou et al. (2017) [13]	CS	32,296	China	16–45 (28.79 ± 2.80)	<28	Mobile communication base station, mobile phone, microwave oven, induction cooker, electric blanket, and hair perming and dying appliance	There was no excess risk of spontaneous abortion in pregnant women using mobile phone (*p* = 0.918), microwave oven (*p* = 0.334), induction cooker (*p* = 0.55), electric blanket (*p* = 1), and hair perming and dying appliance (*p* = 0.539)	8
							The residence within 100 m of mobile communication base station was an independent risk factor for spontaneous abortion (*p* < 0.05) (OR: 0.34; 95% CI: 0.23–0.49)	
Li et al. (2017) [27]	Cohort	913	California	18≤	<10	EMDEX Lite meter (Enertech Consultants Inc.) for 24 h during pregnancy	Women who were exposed to higher MF levels (higher than three quartiles) had 48% greater risk of miscarriage (hazard ratio [HR] = 1.48, 95% CI: 1.03–2.14) than those with lower MF exposure in the lowest quartiles (OR: 1.54; 95% CI: 2.38–1.03)	8
Abad et al. (2016) [28]	CS	413	Iran	18–35 (28.2)	<12	All wireless services, TV Band I, FM-Radio, mid wave, paging, Band III (DVB-T), trains, Band IV (DVB-T), Band V (DAB), GSM-R, GSM 900, L-Band (DAB), GSM 1800, DECT, UMTS-TDD, UMTS DL, W-LAN, and ISM.	An increased risk of miscarriage was observed in women who were exposed to significant levels of electromagnetic wave; however, Wald test did not confirm this finding (OR = 1.12 [0.83–1.52]; *p* = 0.447)	9
Mahmoudabadi et al. (2015) [29]	CC	472	Iran	18–35	<14	Mobile phones	A significant association was observed between use of mobile phone during pregnancy and the risk of spontaneous abortion (*p* < 0.001, OR: 1.11 [1.07–1.16])	7
Wang et al. (2013) [30]	Cohort	450	China	15–44	8	High-voltage power lines and substations (110–500 kV)	Miscarriage risk was significantly associated with maximum alley exposure (*p* = 0.001, OR: 1.78 [1.11–2.82])	8
Shamsi Mohammadabadi (2013) [31]	CC	116	Iran	18–35	<14	EMF in the participants´ houses by an exposure level tester (3D EMF tester/Model: ELF-828; Taiwan)	The magnitude of ELF-EMF in the participants´ houses was significantly different between the two groups (miscarriage and normal delivery) (*p* < 0.001, OR: 1.85 [1.38–2.47])	8
Zhang et al. (2011) [12]	CC	552	China	24–35	9.3 ± 2.8	Computer and cell phone	Pregnant women who used computer and cell phone more than 6 h per week were more likely to have abortion (*P*: 0.05, OR: 1.62 [1.07–2.47])	8
Fucic et al. (2008) [32]	Cohort	112	Republic of Croatia	15–44	<26	X-rays in radiological hospital and radioisotopes in nuclear medicine and biochemical diagnostics hospital	At least a three-fold higher rate of spontaneous abortions was found among women exposed to radioisotopes than those exposed to X-ray (OR = 3.68, 95% CI = 1.39–9.74, *p* < 0.01)	7
Li et al. (2002) [33]	Cohort	969	United States	<35	<10	Spot measurements were taken in the subject’s bedroom, the kitchen, and the most frequently occupied room that was neither a bedroom nor a kitchen	Prenatal maximum MF exposure above a certain level (possibly around 16 mG) may be associated with miscarriage risk (RR: 1.88 [1.2–2.7])	8
Lee et al. (2002) [34]	CC	713	California	≥18	<20	Residential wire codes, very high current configuration, ordinary high current configuration, and ordinary low current configuration	The maximum personal MF exposures is associated with the risk of clinical miscarriages and there was a dose response effect with an increase in exposure and with the number of environments (OR: 1.24 [0.70–2.10])	7
							A prospective substudy showed that mothers exposed to EFMs greater than 2 mG had a 3.58 times (0.89–11.75) increased relative risk of miscarriage compared to mothers exposed to EFMs lower than 2 mG	
Lee et al. (2000) [35]	CC	5,144	California	≥18	≤13	Electric bed heaters	Women who used electric blankets at low settings for most of the night (*N* = 171) had lower risks of spontaneous abortion than who did not use (OR = 0.81, 0.61–1.21)	7
Grasso (1997) et al. [36]	CC	1,656	Italy	15–44	12	Video display units	There was no association between video display terminal (VDT) exposure and spontaneous abortion (OR: 1.01 (0.80–1.21))	7
Juutilainen et al. (1993) [17]	CC	191	Finland	29 ± 4.3	≤12	MF in the living room, in the kitchen, and in the parents’ bedroom	Exposure to MF greater than 2.5 mG or 0.2 A/m in the case group had risks 5.44× higher than the controls (OR = 5.44, 1.10–28.0)	7
Ouellet-Hellstrom et al. (1993) [37]	CC	3,322	United States	20<	<28	Radio and microwave-frequency electromagnetic radiation	The OR in physiotherapists exposed to microwave diathermy equipment (20 or more exposures/month) was 1.28. The overall OR was slightly lower after it was controlled for prior fetal loss (OR = 1.26, 95% CI: 1.00–1.59). The risk of miscarriage was not associated with the reported use of shortwave diathermy equipment (OR = 1.07, 95% CI: 0.91–1.24)	7
Lindbohm et al. (1992) [38]	CC	585	Finland	20–35	≤12	VDTS	Use of VDTs by pregnant women during the first trimester of pregnancy were not a risk factor for spontaneous abortion (OR: 93; CI: 063–1.38; *p* = 0.73)	7
							Workers who had used VDTs for more than 20 h per week compared to those who used VDTs for less than 20 h per week had a 2.1-fold increased risk for miscarriage (3.70–0.80) and based on the type of device used, workers exposed to EMFs more than 0.4 µT had a 3.8-fold increased risk of miscarriage compared to those exposed to EMFs lower than 0.4 µT (1.10–6.80)	
Goldhaber et al. (1988) [39]	CC	1,078	California	17<	<28	VDTS	Exposure over 20 h per week was significantly associated with a relative risk for miscarriage (OR: 1.8; 95% CI: 1.2–2.8)	7

EMF: electromagnetic field; GA: gestational age; QS: quality score; CC: case-control; CS: cross-sectional; OR: odds ratio; CI: confidence interval; EAS: electronic article surveillance; MF: magnetic field.

## 7 Publication bias

Based on the results of Egger’s test, no significant publication bias was observed for the association between EMF and miscarriage (coefficient: 0.07; *p*: 0.113), indicating that the result of meta-analysis was robust 

**Table 3:** Result of univariate meta-regression analysis for heterogeneity determinants of ORs of miscarriage

Variables	Coefficient	95% CI	*p*-value
Mother’s age (year)	0.051	−0.133 to 0.236	0.537
Publication year (year)	−0.011	−0.032 to 0.010	0.300
Sample size (number)	−10 × 10^5^	−19 × 10^5^ to 1 × 10^5^	0.030*

*: significance; CI: confidence interval.

## 8 Discussion

Our results demonstrated that the risk of abortion was increased by 1.27× for pregnant women exposed to ELF and intermediate frequency EMFs in the first trimester of pregnancy. This may be due to living in urban areas, such as living on the outskirts of a city and living near electricity or cell towers, and the long-term use of mobile phones and VDTs.

Because the effects of EMF exposure on pregnant women are different, the WHO has recommended need for more epidemiological studies to investigate the association between exposure to EMF and abortion [30]. There are three sources, such as equipment for medical diagnosis and treatment [37,40], VDTs [36,39,41] and electrical appliances and devices [13,26,29,35], and high voltage power tower near residences, that are producers of EMFs [17,30,33,34], Which are discussed in order. Ionizing radiation is one of the most important waves based on wavelength, and majority of personnel working in the industries and diagnostic and therapeutic departments are exposed to it. Ionizing radiation and certain metals have been known to cause the spontaneous abortions in humans and fetal death in animals [2,42]. In this regard Fucic et al. reported that the occurrence of miscarriages in women exposed to radioisotopes were at least three times more than that in women exposed to X-ray. In addition, chromosome aberrations in peripheral blood lymphocytes were increased in radiation-exposed group compared to the X-ray-exposed group [32]. Waves between 27 and 250 MHz are being used for treatment purposes, because they increase body temperature by 1–2°C by absorbing energy [28]. Physiotherapists experience the exposure to EMFs from shortwave and microwave diathermy devices. This wave can penetrate the adipose tissue and bone and are easily absorbed into the blood and muscle. In fact, the special feature of absorption of RF and MF in the muscle with the aim of improving the blood flow to the deep muscle tissues is the basis of medical diathermy. However, microwaves are selectively absorbed in tissues with high water content such as muscle, blood, and amniotic fluid compared to shortwave radiation. In this regard, the study result showed that use of shortwave diathermy by female physiotherapists was not associated with the risk of miscarriage [43]. However, another study showed that the risk of miscarriage in the group with highest exposure (≥20 exposures/month) to microwave diathermy equipment was increased by 59% [37]. Also, exposure to shortwaves was related to considerably increased OR for congenital malformations and low birth weight [44].

Exposure to MFs non-ionizing radiation is considered as a serious public health challenges. MFs can be produced from older equipment with low frequency MFs (power lines, appliances, and transformers) and from newer sources with higher frequency MFs (wireless networks, cell towers, wireless devices like cell phones, etc.). Cell phone RF waves may result in DNA damage in the fetus [13] and microwave radiation may damage the placental barrier. Results of a study showed that there was an association between cell phone use by mothers and an increased risk of miscarriage, congenital anomalies, and behavioral problems in children [9]. The radiation of mobile phone base stations installed within 100 m of the residence may not have much effect on the general population, but may adversely affect the chromosomal structure of the fetus. An animal study showed that by placing the cell phone in the middle of the incubator for 10–15 days increased the growth restriction of the fetus in exposed group. The pulsed current EMFs may cause DNA damage of the fetus, leading to increased micronucleus rate [45].

The type of EMF in cell phones is both ELF-EMF and RF-EMF. To investigate cell phone effects, the location of the cell phones when not in use, use of hands-free equipment, use of phones for other applications, the specific absorption rate [46] reported by the manufacturer and the average of the effective specific absorption rate (SAR) (average duration of calling time per day × SAR) were measured [31]. The underlying mechanisms of the effects of EMFs on the risk of spontaneous abortions are not well understood. An adverse effect during early fetal development at the cellular level by EMFs of cell phones can conceivably lead to fetal death. Based on the device distance from outside the body to inside the uterus, the exposure of the fetus is likely to be ELF electromagnetic radiation. But perhaps a particular window of frequency, waveform, and intensity of low frequency EMFs can be destructive. In this regard, Shamsi et al. showed that the risk of spontaneous abortion was increased in woman who were exposed to ELF-EMF during pregnancy [31]. However, estimates of the association between cell phone use and the risk of miscarriage did not change significantly despite the adjustment of many known or suspected risk factors. A study conducted on the relationship between the waves generated by a BTS antenna model and spontaneous abortion showed that women exposed to very high-intensity EMFs had an increased risk of miscarriage [28].

Other devices which are commonly used in shops and air terminals are the EAS systems with intermediate frequency (IF) MFs. Accordingly, the results of a study showed that cashiers working in supermarkets with EAS devices at the frequency of 8.2 MHz did not report any increase in the abortion, preterm birth, and low birth weight compared to those working in small supermarkets without such systems. This may be due to the fact that the frequency of EMFs has ELF which is emitted from various electronic devices near the sellers of both stores, and they mainly produce 50 Hz MF, and their share cannot be eliminated. In fact, cashiers working in supermarkets with EAS devices are more exposed to static MFs and intermittent IF MFs, while smaller store cashiers are more exposed to ELF-EMF, so no significant differences on pregnancy outcomes were observed between the two groups [26].

In a study conducted on pregnant women with gestational age less than 10 weeks where their exposure to EMFs were measured by a special monitor, it shows that these women exposed to more than 16 mG of EMFs had 80% increased risk of abortion compared to other pregnant women who were exposed to less than 16 mG of EMF [47]. The theories that empower the possible association between EMF exposure and abortion are as follows: (1) the chemical interaction in cell membrane, (2) reduction in permeability in cell membrane and consequent decrease in cellular connections, (3) increase in free radicals, (4) disorder in mitotic divisions, and (5) damage to cellular proteins and cellular disconnection [48]. The other theory about the association between EMF exposure and abortion is that the depth of penetration of the waves increases in tissues with less water [47].

Another electronic device that is widely used in urban communities is electrically heated beds or ceiling heating systems that produce ELF-EMFs. A prospective study showed that electric blanket use at conception was associated with an increased risk of spontaneous abortion, but heated water bed use was not related to an increased risk of spontaneous abortion at conception. Women living in homes classified as “very high” or “ordinary high” current configuration were not at greater risk [49]. In this regard, the results of another study on fetal loss in families living in homes with or without ceiling cable electric heater showed that the electrically heated beds with 60 Hz EMF exposure may influence human fetal development directly, or through an effect on the mother. In addition, they analyzed the fetal loss during the season. In fact, the difference between user and non-user groups was not examined, but the difference between user groups with the highest exposure compared to those with the least exposure during the month was investigated. There was a significant correlation between the monthly fetal loss rate and the increase in daily temperature in homes with ceiling cable heater, but not in other homes. Using this method, they found that each user group reported fetal loss disproportionately often during the season when EMF exposure was increased [50]. Contrarily, in a cohort study, Lee et al. showed that the rates of spontaneous abortion were lower in women who used electric bed heaters as compared with non-users. Users of electric blankets at low settings for most of the night had lower risks of spontaneous abortion compared to non-users. But for 20 women who used electric blankets at high setting for 1 h or less, the rate was different. The difference in OR in the above two studies may be due to how users use low settings versus high settings and climatic differences in the two studies. The colder the weather, more users use the high settings of the device, thus disconnecting and reconnecting the device. But in the study by Lee et al., most of the electric blanket users kept their blankets at low settings for most of the night, which did not have on or off cycle, resulting in no MF. Therefore, in the study, the use of electric blankets may not reflect exposure to nighttime MFs. Other studies have shown an association between the 60 Hz MF exposure and its effect on immature human beings [51].

VDTs are one of the most widely used devices. Research has shown that the amount of ionizing radiation emitted by VDTs can be detected with equipment for the standard radiology. VDTs generate very low-frequency MFs from the fly back transformers. The results of a case-control study suggested a significantly increased risk of miscarriage for working women in the first trimester of pregnancy who reported using VDTs for more than 20 h/week compared to other working women who reported not using VDTs. One possible explanation for these results is that women who had adverse pregnancy outcomes may have over reported their exposures to VDTs and/or women with normal births may have underreported theirs. The results may also be due to unmeasured factors confounded with high VDT use like poor ergonomic conditions or job-related stress [39]. However, another study measuring the EMFs from VDT showed that the OR for spontaneous abortion in female employees working with VDTs was not increased. However, the OR with adjustment confounder factors (ergonomic factors and mental work load factors) for employees who had used a VDT with a high level of ELF-EMF (>0.9 µT) was increased 3× in comparison with those using a terminal with a low level of these MFs (<0.4 µT) [38]. Schnorr et al. showed that the female telephone operators who used VDTs at work were not associated with an increased risk of spontaneous abortion compared with operators who did not use VDTs. Abdominal exposure to ELF-EMF was similar for both operators who used VDTs and those who did not [52]. It should be noted, that in this study to estimates of exposure, they. measured EMF at one VDT work station and abdominal exposure to ELF-EMF in female telephone operators who used VDTs was lower than above study. One of the weaknesses of the study was recall bias. Concerns have also been expressed by users that VDTs may generate the hazardous electromagnetic radiation, but this study measuring only two models of VDTs showed that the abdominal exposure to ELF-EMF (45–60 Hz) for operators who had used VDTs was similar to the range of exposure to MF in homes. The researchers’ conclusions showing that there is no association between the MF from VDTs and spontaneous abortion are criticized. This may be due to not taking into consideration subclinical abortions and the incorrect definition of exposure (exposure to VDTs was limited to time working with the device, while the highest non-ionizing radiation comes from the back or even the top of the device). Therefore, ELF-EMF measurements at the work stations may provide more information about spontaneous abortion.

Other problems of urban life are suburbanization and residence near high-voltage power towers. To obtain a more accurate estimate of the exposure of pregnant women to EMFs, the following studies have evaluated the exposure to EMFs using different measurement tools. In this regard, the findings of a study showed a strong association between the residential exposure to ELF-MFs (at the intensity of greater than 50 Hz) and fetal loss in early pregnancy with a relative risk of 3–5× in the highest exposure group (over 0.2 or 0.5 µT). The high OR in MFs with a strength of greater than 0.5 A/m or an average residence with a MF strength higher than 0.2 A/m indicates that MF above this value may adversely affect the fetal growth. Retrospective MF measures may lead to degree of misclassification of exposure groups [17]. A nested case-control study was conducted to assess exposure to EMFs. Wire codes, area measures, and three personal meter metrics were assessed. Adjusted OR of the time-weighted average of pregnant women exposed to ELF-EMF was 1.7. For the top three quartile, starting with the highest quartile, 2.3, 1.9, and 1.4 were the maximum values, respectively. In the prospective study, the time-weighted average of pregnant women exposed to ELF-EMF over 2 mG was associated with a three-fold increased relative risk of miscarriage [5]. Juutilainen et al. have reported that indoor exposure above 2.5 mG was associated with a five-fold increase in the miscarriage [17]. Another study showed that a 2.3-fold increase in the relative risk of miscarriage among the siblings in case group, where families live in homes with MFs above 0.2 μT (2 mG), compared to control group, where families live in homes with MFs less than 0.2 μT. It should be noted that EMFs were measured only in 17% of the case group and 34% of the control group. However, with the aggregation of siblings in the case and control groups, the risk of miscarriage was not associated with the measured MF [53]. Another study recruited the pregnant women to wear an ACTi graph accelerometer and EMDEX MF monitor for 7 days and found a positive association between level of activity and likelihood of elevated exposure, most strongly for cut-points of 16 or 20 mG, for both working and nonworking women among whom ORs in the uppermost quartile ranged from 2.1 to 2.6. In fact, peak MF exposure was associated with increased risk of miscarriage [54]. A 2-year cohort study did not find significantly increased risk of miscarriage to be related to the average front-door exposure. However, miscarriage risk was significantly associated with maximum alley exposure. The relative risk of miscarriage was 2.35 [30].

A study was carried out on 149 pregnant women seeking induced abortion of unwanted pregnancies. The women were asked to wear an EMDEX Lite MF meter for a 24 h period to measure MF exposure level. The results showed that embryonic bud length was inversely correlated with maternal daily MF exposure level. Logistic regression showed that women with her 75th percentile of daily MF measurements ≥0.82 mG had a 3.95-fold risk of having a fetus with a shorter embryonic bud length than those whose daily MF exposure were <0.82 mG. MF exposure was correlated with a higher degree of apoptosis. One of the most important strengths of the study is the use of EMDEX Lite, which objectively showed the MF exposure level from various sources [55]. Another study demonstrated that pregnant women who were exposed to higher MF levels had 2.72× the risk of miscarriage than those with lower MF exposure [27], so that the peak MF exposure with risk ratios of 1.8 (with a threshold of approximately 16 mG) as measured by personal monitoring in early pregnancy may be associated with miscarriage risk [32]. These findings are consistent with previous studies examining the association between high MF exposure and miscarriage risk [29,30,46].

### 8.1 Limitations

Most of the studies were retrospective and few studies were prospective. One of the potential problems of retrospective studies is the recall bias and overexpression of exposure, which affects the accuracy of information. In addition, prospective studies have not been associated with precise control of interfering factors such as chromosomal aberrations, socioeconomic factors, and job stress. Most of the studies conducted on animals have shown an association between abortion and the EMF exposure, while the amount of waves exposed is much higher than the daily exposure of human societies, which cannot be generalized to human studies, and none of the studies has precisely addressed the mechanism of action. It is recommended that prospective studies be conducted with a larger sample size and taking into account the marginalized women of industrial cities with more control over confounding factors and by accurately measuring the daily EMF exposure in mothers.

## 9 Conclusion

Exposure to EMFs is associated with 1.27× increased risk of abortion. The maximum ELF-MF exposure greater than 50 Hz or 16 mG or even 2–2.5 mG is associated with an increased risk of abortion. Considering the limited evidence base, it may be prudent to advise women against this potentially important environmental hazard. Indeed, pregnant women should receive tailored counseling and stimulate more the much needed research.
